# Beyond national fertility rates: unmasking territorial differences in adolescent pregnancy in Ecuador

**DOI:** 10.3389/frph.2026.1863795

**Published:** 2026-07-22

**Authors:** Mónica Izurieta-Guevara, Erika Lozano-Erazo, Sami Sánchez-Verduga, Cristina Núñez-Vásquez, Lorena Izurieta-Guevara, Emmanuelle Quentin

**Affiliations:** 1 Centro de Investigación en Salud Pública y Epidemiología Clínica (CISPEC), Facultad de Ciencias de la Salud Eugenio Espejo, Universidad UTE, Quito, Ecuador; 2 Observatorio de Género Universidad UTE, Quito, Ecuador; 3 Hospital AXXIS de Especialidades, Servicio de Ginecología y Obstetricia, Quito, Ecuador

**Keywords:** adolescent pregnancy, adolescent fertility, Ecuador fertility rates, spatial epidemiollogy, territorial inequality, early unions

## Abstract

**Introduction:**

Teenage pregnancy is a significant public health challenge that may contribute to cycles of poverty in the context of social inequality. In Latin America and the Caribbean, Ecuador ranks third in terms of fertility rates. The objective of this study is to describe temporal trends and territorial patterns of adolescent fertility in Ecuador between 2015 and 2024, differentiating early adolescents (10–14 years) and late adolescents (15–19 years), and identifying priority territories for public health monitoring.

**Method:**

A descriptive observational study was conducted using anonymized databases from the National Institute of Statistics and Census (INEC). Fertility rates were calculated for two groups: early adolescents (10–14 years) and late adolescents (15–19 years). Data were analyzed by province, area of residence, and social variables. Monotonic temporal trends were assessed using the non-parametric Mann–Kendall test.

**Results:**

The national fertility rate declined steadily, from 40.75 per 1,000 (95% CI: 40.44–41.08) in 2015 to 20.53 in 2024 (95% CI: 20.30–20.75). Despite this overall progress, geographic heterogeneity in fertility rates was observed, with the highest rates and slowest declines concentrated in Amazonian and Coastal provinces. Fertility rates remained consistently elevated among adolescents in early unions, those with lower educational attainment, and within Indigenous and Mestizo populations. Mann–Kendall analyses identified priority provinces with non-significant downward trends, particularly among early adolescents.

**Conclusion:**

The study identified declining adolescent fertility rates alongside persistent territorial disparities across Ecuador. Differences observed by province, age group, ethnicity, educational attainment, and early unions underscore the importance of complementing national indicators with disaggregated analyses. This perspective may contribute to a more nuanced understanding of adolescent fertility patterns and may guide territorial surveillance and public health planning.

## Introduction

Adolescent pregnancy is a global problem with serious implications for health, development, and the economic costs of a region. Teen pregnancy refers to pregnancy in girls and adolescents aged 10–19 (1). Each year, in low- and middle-income countries (LMICs), more than 21 million adolescents aged 15–19 become pregnant, and one million girls (10–14 years) give birth (2, 3). Although there has been a global decline in adolescent fertility over the past decade, Latin America and the Caribbean still have higher fertility rates than other continents. According to 2022 data, the region recorded between 51 and 52 births per 1,000 adolescents (15–19 years), higher than the global rate of 39. Thus, Latin America and the Caribbean are the regions with the second-highest rate of pregnancy among girls (10–14 years) and adolescents (15–19 years), followed by sub-Saharan Africa (4). Pregnant adolescents face up to three times the risk of maternal mortality compared with adult women and are forced to resort to unsafe abortions (2). Complications from pregnancy and childbirth are the leading cause of death among adolescents aged 15–19 worldwide (5).

Adolescent pregnancy may reflect a country's social inequalities and disproportionately affect vulnerable social groups and ethnic minorities (6–8). Early motherhood entails high economic costs for both women and States in Latin America and the Caribbean. The combined annual costs for 15 countries in this region total US$15.357 billion, of which 88.2% falls on women due to opportunity costs associated with education, employment, inactivity, and labour income, while 11.8% affects the State through healthcare expenses and lost tax revenue (9).

Ecuador has the third-highest adolescent fertility rate in Latin America, after Nicaragua and the Dominican Republic (2). According to the National Institute of Statistics and Census, in 2024, Ecuador's adolescent fertility rate reached 39.53 births per 1,000 women (8). Although the trend is downward, it remains significantly higher than in high-income countries; for example, it is twice as high as in the United States and six times higher than in Spain.

In this context, the objective of this study is to describe temporal trends and territorial patterns of adolescent fertility in Ecuador between 2015 and 2024, differentiating early adolescents (10–14 years) and late adolescents (15–19 years), and identifying priority territories for public health monitoring. Previous studies in Ecuador have described adolescent pregnancy at the national level, investigated social and demographic predictors, or examined only specific local contexts (10–12). However, there remains a lack of evidence assessing long-term subnational fertility trends across provinces, distinguishing between early (10–14 years) and late (15–19 years) adolescents, and identifying priority territories through temporal trend analyses. This study addresses these gaps by integrating national data from 2015 to 2024 and applying spatial and temporal analyses to examine subnational variation and guide territorial prioritization for public health monitoring. By uncovering these differences, the study provides evidence for the design of targeted public policies on adolescent pregnancy and the implementation of preventive strategies.

This issue is linked to the 2030 Agenda for Sustainable Development and has cross-cutting implications for SDG 3 (good health and well-being), SDG 5 (gender equality, including the elimination of all forms of violence against women and girls), and SDG 1 (ending poverty), as adolescent pregnancy is considered a process that contributes to the intergenerational transmission of poverty (13).

## Materials and methods

This study is a descriptive ecological analysis based on publicly available databases from the National Institute of Statistics and Census (INEC). These databases contain no fields that allow individual identification. The primary sources were the Statistical Registry of Live Births and official population estimates and projections. This design was used to characterize the territorial patterns of adolescent fertility and their evolution over ten years.

All annual datasets were merged into a single database and then filtered according to the following inclusion criteria: births occurring between 2015 and 2024 (the most recent decade available), mothers aged 10–19 years at the time of delivery, and mothers residing in Ecuador. Population estimates and projections were restricted to females aged 10–19 years and categorized into two groups: girls (10–14 years) and adolescents (15–19 years).

The primary indicator analyzed was the fertility rate, defined as the number of live births to adolescent mothers per 1,000 adolescent females in the population. For the age-specific rate, the number of live births to women in a specific age group was divided by the total number of women in that group and multiplied by 1,000.

Statistical summaries and graphical representations were generated for each year, province, and five-year age group (10–14 and 15–19 years).

Exact 95% confidence intervals (95% CI) for fertility rates were estimated assuming a Poisson distribution for the observed number of births.

Monotonic temporal trends in age-specific fertility rates were assessed using the Mann–Kendall test, a rank-based non-parametric method appropriate for this analysis for three reasons: it does not assume a normal distribution of the outcome variable, it is robust to outliers and missing values, and it is well-suited to detecting consistent directional change in short-to-moderate time series without requiring linearity. The direction and magnitude of each trend were quantified using Kendall's tau (*τ*), which ranges from −1 to +1. A value of −1 indicates a perfectly consistent decreasing trend across all time points, while a value of +1 indicates a perfectly consistent increase; values near zero indicate no discernible temporal pattern. Statistical significance was assessed at *α* = 0.05. Given the exploratory and descriptive nature of this analysis — conducted separately per province and age group to characterise spatial heterogeneity in trend direction — formal correction for multiple comparisons was not applied; non-significant results (*p* ≥ 0.05) were retained in the dataset but excluded from maps and interpreted with caution. Results should therefore be interpreted as indicative of spatial patterns rather than as confirmatory evidence of trend in individual provinces.

Only provinces with a statistically significant negative *τ* were mapped, as these represent jurisdictions where fertility rates declined consistently over the study period. The colour scale aligns with this interpretation: lighter red tones correspond to *τ* values closer to −1 (stronger, more consistent declines in adolescent fertility), while darker red tones correspond to *τ* values closer to 0 (weaker declines in adolescent fertility). Although a steeper decline might superficially appear more favourable, this scaling was intentionally designed to draw attention to provinces with weaker reductions (*τ* near 0), as these represent areas where adolescent fertility rates have been most resistant to change — and therefore where the public health burden may be most persistent. Provinces with non-significant trends were displayed in grey, indicating insufficient evidence of a consistent directional change over the study period.

As a complement to the study, social variables related to union status, education, and ethnicity – coming from the most recent Ecuadorian Census conducted in 2022 – were explored to recommend future studies with a social determinants approach.

Full provincial reports, including Mann-Kendall tau, Sen's slope, and associated *p*-values, are reported in the datasheet (supplementary material).

Analyses were conducted using R (version 4.6.0) in RStudio (Posit Team). The Kendall package was used to perform the Mann–Kendall test for monotonic trends, and the trend package was used to compute Sen's slope as a complementary estimate of the magnitude of temporal change. Spatial analysis and mapping were carried out using the sf and ggspatial packages.

## Results

In the final year of the period studied, early adolescents (10–14 years) and late adolescents (15–19 years) accounted for 25% of all women of childbearing age (10–49 years). For analytical purposes, the results presented below are interpreted by distinguishing between two age groups: pregnancy in early adolescents (10–14 years) and pregnancy in late adolescents (15–19 years).

### Adolescent fertility by age group

Fertility among early and late adolescents in Ecuador from 2015 to 2024 shows a sustained decline. The rate fell from 40.76 per 1,000 in 2015 (95% CI: 40.44–41.08) to 20.53 per 1,000 in 2024 (95% CI: 20.30–20.75). Overall, the results indicate a reduction in adolescent fertility in the country (Table 1).

**Table 1:** National trend in total adolescent fertility (10–19 years), Ecuador, 2015–2024.

Year	Births	Female population (10–19 years)	Fertility rate per 1,000 adolescents	95% CI
2015	62,374	1,530,321	40.76	40.44–41.08
2016	56,372	1,534,816	36.73	36.43–37.03
2017	57,273	1,540,103	37.19	36.88–37.49
2018	56,490	1,548,290	36.49	36.19–36.79
2019	52,043	1,556,976	33.43	33.14–33.71
2020	45,650	1,560,746	29.25	28.98–29.52
2021	41,786	1,561,382	26.76	26.51–27.02
2022	40,290	1,562,796	25.78	25.53–26.03
2023	36,470	1,565,209	23.30	23.06–23.54
2024	32,184	1,567,881	20.53	20.30–20.75

The differences by age group and region are presented below:

**Early adolescents**
  In this group, there is an overall decline in the fertility rate between 2015 and 2024, from 3.38 births per 1,000 adolescents (95% CI: 3.25–3.51) in 2015 to 2.04 per 1,000 (95% CI: 1.94–2.14) in 2024. This represents an approximate reduction of 39.6% over the period analyzed. Although the decrease was sustained in most years, slight temporary increases were observed in 2017 and, especially, between 2021 and 2022, when rates rose from 2.14 (95% CI: 2.04–2.25) in 2020 to 2.38 (95% CI: 2.27–2.49) and 2.46 (95% CI: 2.35–2.57) per 1,000, respectively. The 95% confidence intervals are relatively narrow across all years, indicating precise estimates (Table 2).
**Late adolescents**
  Among adolescents aged 15–19, the fertility rate declined between 2015 and 2024. It fell from 78.40 births per 1,000 adolescents in 2015 (95% CI: 77.77–79.03) to 39.53 per 1,000 in 2024 (95% CI: 39.09–39.97), representing an approximate reduction of 49.6% over ten years. In contrast to the 10–14 age group, no significant increases were observed in recent years; instead, the downward trend was continuous from 2018 onwards (Table 3).

**Table 2:** National trend in fertility among girls aged 10–14 years, Ecuador, 2015–2024.

Year	Births	Female population (10–14 years)	Fertility rate per 1,000 adolescents	95% CI
2015	2,593	767,812	3.38	3.25–3.51
2016	2,284	770,659	2.96	2.84–3.09
2017	2,327	774,266	3.01	2.88–3.13
2018	2,116	778,910	2.72	2.60–2.83
2019	1,837	783,835	2.34	2.24–2.45
2020	1,689	787,733	2.14	2.04–2.25
2021	1,881	790,727	2.38	2.27–2.49
2022	1,948	793,224	2.46	2.35–2.57
2023	1,679	794,799	2.11	2.01–2.22
2024	1,620	794,636	2.04	1.94–2.14

**Table 3:** National trend in fertility among adolescents aged 15–19 years, Ecuador, 2015–2024.

Year	Births	Female population (15–19 years)	Fertility rate per 1,000 adolescents	95% CI
2015	59,781	762,509	78.40	77.77–79.03
2016	54,088	764,157	70.78	70.19–71.38
2017	54,946	765,837	71.75	71.15–72.35
2018	54,374	769,380	70.67	70.08–71.27
2019	50,206	773,141	64.94	64.37–65.51
2020	43,961	773,013	56.87	56.34–57.40
2021	39,905	770,655	51.78	51.27–52.29
2022	38,342	769,572	49.82	49.33–50.32
2023	34,791	770,410	45.16	44.69–45.64
2024	30,564	773,245	39.53	39.09–39.97

**Figure 1:**
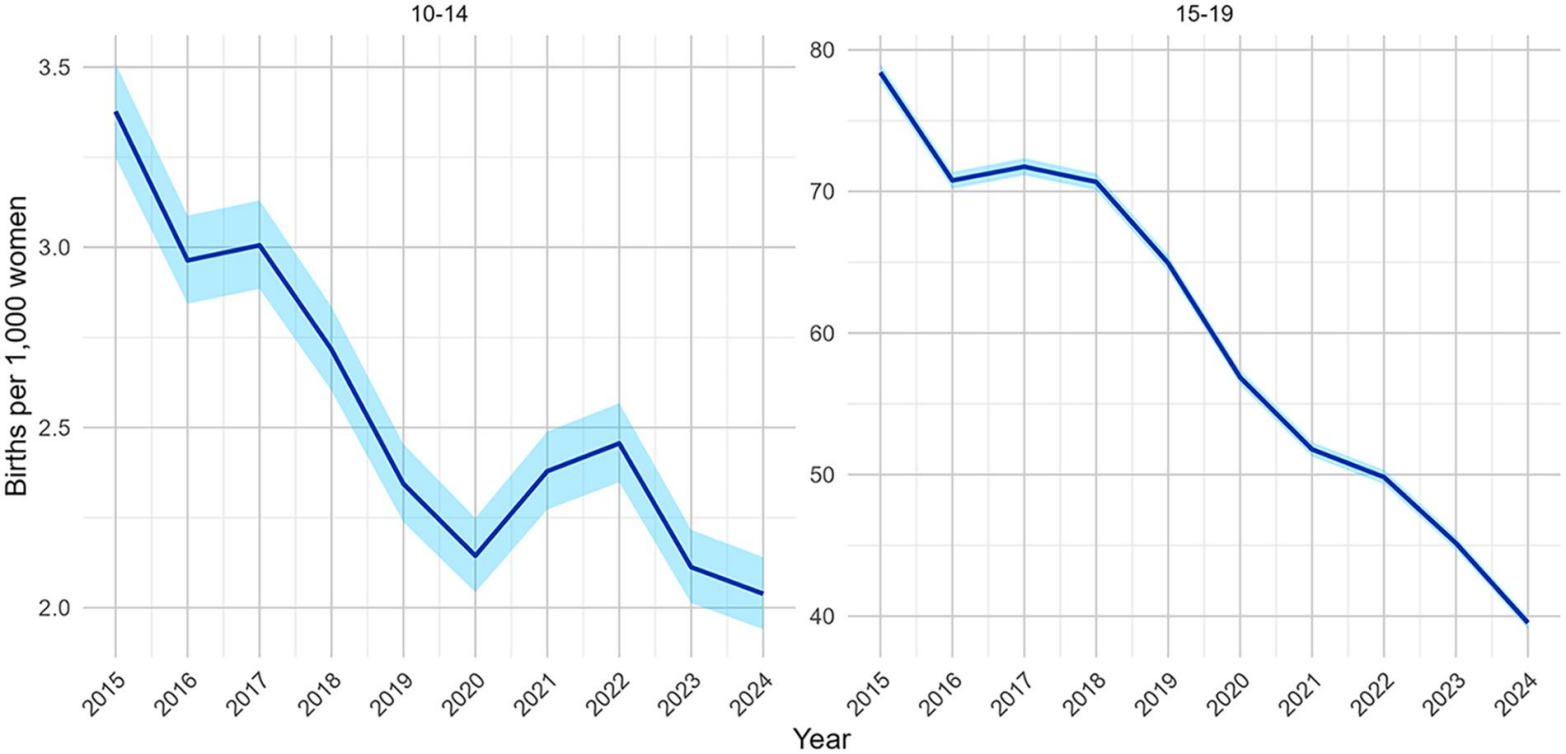
Adolescent fertility rates per 1,000 women among girls aged 10–14 years and adolescents aged 15–19 years in Ecuador, with 95% confidence intervals, 2015–2024.

The trends for both age groups, along with their confidence intervals, are shown in Figure 1.

### Geographical differences in adolescent fertility

At the political-geographical division level in Ecuador, the two age groups analyzed show higher fertility rates in the northern areas bordering Colombia, particularly in some provinces of the Amazon region and the northern Pacific Coast.

**Figure 2:**
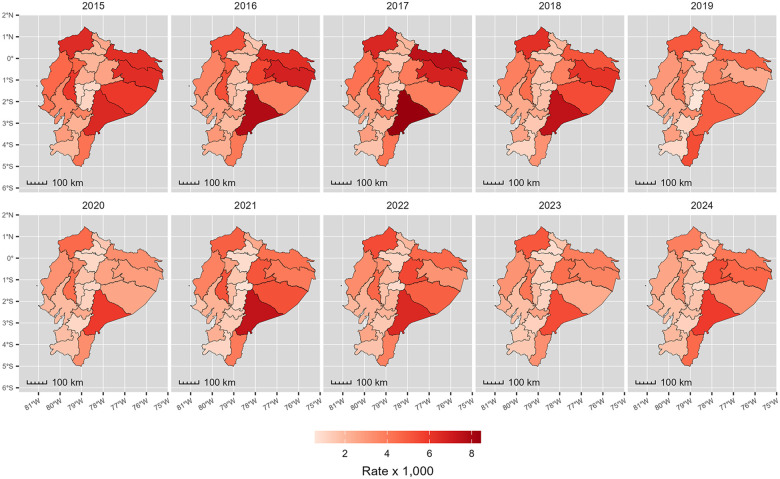
Adolescent fertility rates per 1,000 by province in Ecuador 2015–2024 (ages 10–14).

Among early adolescents (Figure 2), the spatial analysis presents territorial heterogeneity across Ecuadorian provinces during 2015–2024. Overall, the highest rates were concentrated in Amazonian provinces and certain areas of the Pacific Coast, while the central Sierra provinces recorded the lowest values throughout the period. A pattern of high rates was observed in southern and south-central Amazonian provinces, particularly in Morona Santiago. Provinces Sucumbios and Orellana, in the Amazon region, recorded high levels throughout the period. This pattern may suggest a persistent territorial burden of early adolescent fertility in Amazonian regions. In contrast, central Sierra and some Andean provinces maintained low and relatively stable rates, reflected by lighter shades throughout the analyzed years.

**Figure 3:**
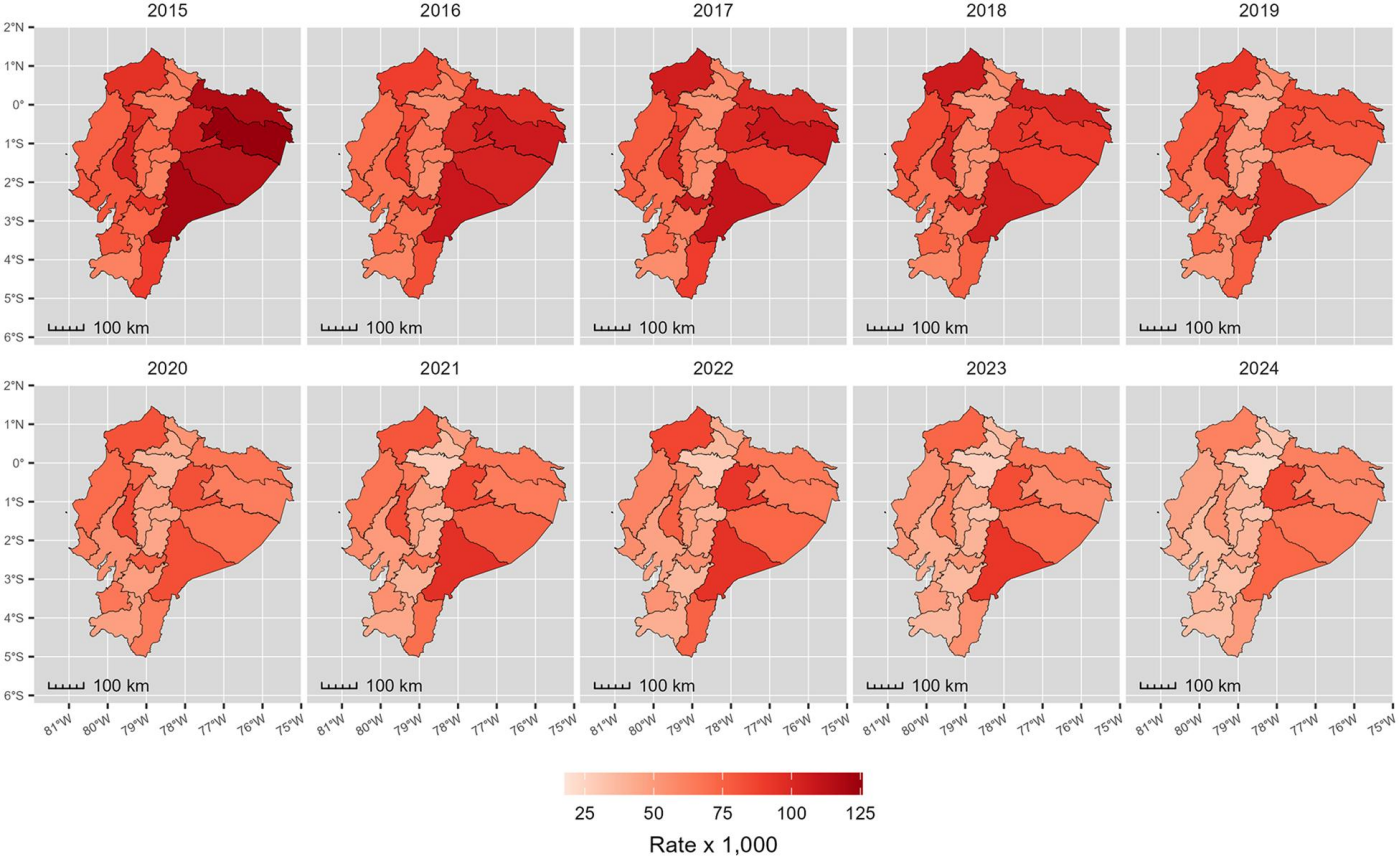
Adolescent fertility rates per 1,000 by province in Ecuador 2015–2024 (ages 15–19).

The analysis of late adolescents (Figure 3) over time shows a gradual, relatively homogeneous decline in rates from 2015 to 2024. Darker shades progressively diminish across almost the entire country, reflecting the national reduction observed in overall rates for the 15–19 age group. However, some Amazonian provinces continue to display relatively high levels even in the final years of the analysis.

Compared with the early adolescent group, the spatial pattern is similar, with higher rates concentrated in the Amazon and lower rates in the central Sierra; however, rates in the 15–19 age group are considerably higher. Additionally, the temporal decline appears more uniform and sustained among adolescents aged 15–19, whereas in the 10–14 age group, more marked fluctuations were observed in certain years and provinces.

### Trends in adolescent fertility rate by province

Geographic analysis of adolescent fertility in Ecuador from 2015 to 2024 identified heterogeneous territorial patterns and temporal trajectories across age groups. Despite the national decline, the results show that this decline has not been uniform across the country, and that certain provinces maintain high levels of adolescent fertility rates throughout the period under review.

### Early adolescents

**Figure 4:**
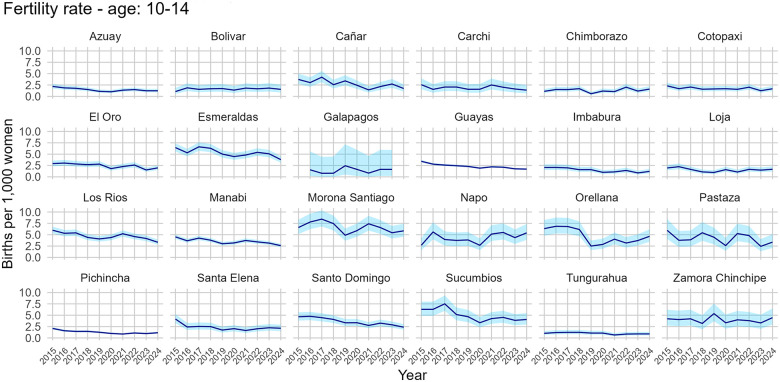
Trends in the adolescent fertility rates per 1,000 by province in Ecuador 2015–2024 (ages 10–14).

Figure 4 shows early adolescent fertility trends (ages 10–14) in Ecuador between 2015 and 2024, revealing a general decline in rates across most provinces; however, the magnitude and consistency of this decrease vary across regions. Amazonian provinces exhibited the highest rates: Morona Santiago, Napo, Orellana, Pastaza, and Sucumbíos consistently reported levels above the national average, with rates in several years exceeding 5 births per 1,000 girls. Among these, Morona Santiago and Sucumbíos stood out for recording some of the highest values at the beginning of the time series. Although these provinces also showed reductions in recent years, their rates remained relatively high compared to other regions of the country. Orellana displayed a particular pattern, with high rates until 2018, followed by a sharp decline and subsequent moderate fluctuations. Pastaza and Napo showed greater year-to-year variability, reflected in both the oscillations of the curves and wide confidence intervals, suggesting less temporal stability.

In the Coastal region, provinces such as Esmeraldas, Los Ríos, Santo Domingo, and Manabí had intermediate-to-high rates at the start of the period, with a gradual decline. Esmeraldas and Los Ríos maintained relatively elevated values compared with Sierra provinces, although there were sustained decreases through 2024.

Provinces in the central and southern Sierra—such as Azuay, Pichincha, Tungurahua, Chimborazo, Loja, and Bolívar—consistently recorded the lowest and most stable rates throughout the period. In these provinces, rates generally remained close to or below 2 births per 1,000 girls, with narrower confidence intervals, indicating greater precision and stability in the estimates. Cañar behaved differently from other Andean provinces, with relatively higher rates and moderate fluctuations during the period.

In general, confidence intervals were wider for early adolescents across several Amazonian provinces, indicating annual variability. In contrast, more populous provinces such as Pichincha and Guayas had narrower intervals and more stable temporal trajectories.

### Late adolescents

**Figure 5:**
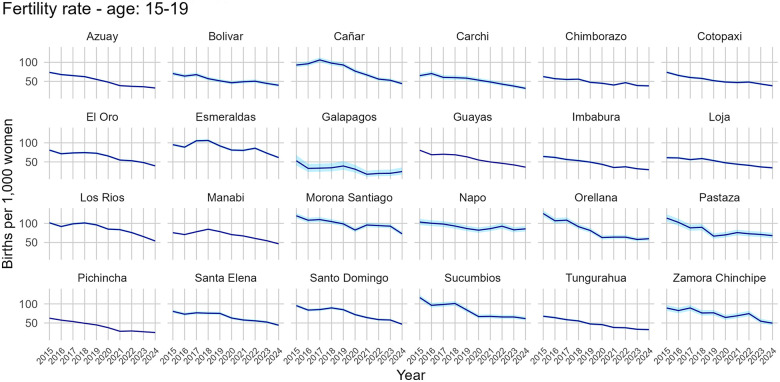
Trends in the adolescent fertility rates per 1,000 by province in Ecuador 2015–2024 (ages 15–19).

The provincial analysis of fertility rates among adolescents aged 15–19 (Figure 5) shows a sustained decline across nearly all provinces in Ecuador between 2015 and 2024, although significant territorial disparities persist in both the magnitude of the rates and the consistency of trends.

Amazonian provinces recorded the highest rates throughout the period. Morona Santiago, Orellana, Sucumbíos, Napo, and Pastaza stood out for maintaining high levels, particularly between 2015 and 2018, when several exceeded 100 births per 1,000 adolescents. While these provinces showed gradual declines towards 2024, they continued to rank among the highest in the country for adolescent fertility. Morona Santiago consistently exhibited among the highest fertility rates throughout the study period and remained among the highest-rate provinces despite declines over time. Sucumbíos and Orellana followed similar trajectories, with marked declines after 2019 but without converging towards the levels seen in Andean provinces.

In the Coastal region, provinces including Esmeraldas, Los Ríos, Manabí, and Santo Domingo also reported high rates, though lower than in the Amazon. These provinces saw relatively steady declines over time, particularly after 2018. Los Ríos and Esmeraldas remained persistently above the national average for much of the period. In contrast, provinces in the central and southern Sierra—particularly Pichincha, Azuay, Loja, Tungurahua, and Chimborazo—showed the lowest and most stable rates.

Compared to the 10–14 age group, the spatial pattern was broadly similar, with higher rates concentrated in the Amazon and parts of the Coast, and lower rates in the central Sierra. However, in the 15–19 age group, rates were substantially higher and territorial differences more pronounced.

### Territorial prioritization

**Figure 6:**
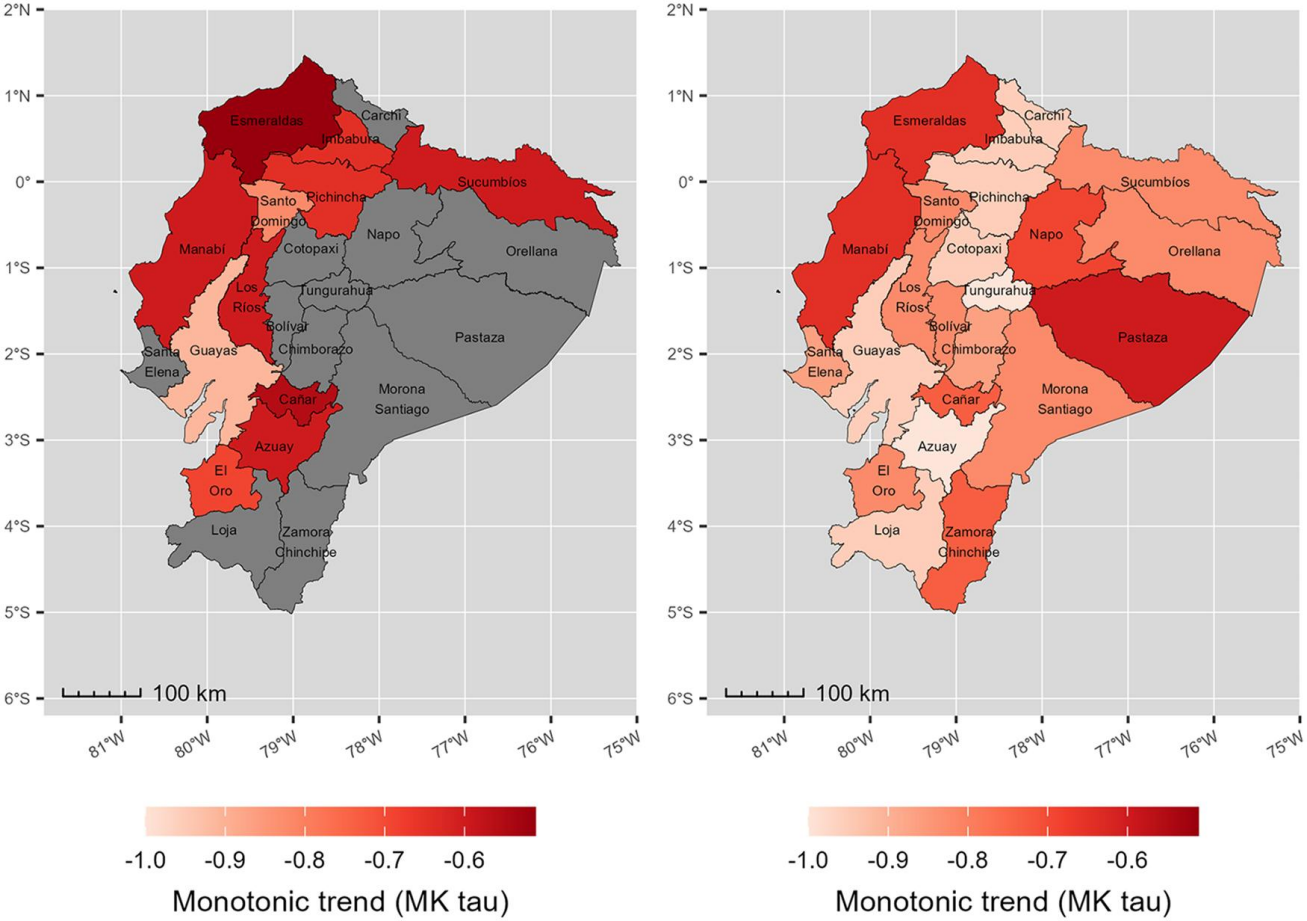
Spatial distribution of monotonic trends in adolescent fertility rates across Ecuadorian provinces, measured using the mann–kendall tau coefficient, for girls aged 10–14 years (left panel) and adolescents aged 15–19 years (right panel), 2015–2024. Darker shades indicate lower decreasing trends in fertility rates. Provinces shown in gray did not present statistically significant trends.

For geographical priorization, monotonic temporal trends were assessed using the non-parametric Mann–Kendall test. A sequential red color palette was used to represent statistically significant negative Kendall's tau values, with darker red tones indicating weaker declines in fertility rates (tau values closer to zero) and lighter red tones indicating stronger declines (tau values closer to −1). This color scaling was intentionally designed to visually emphasize areas with slower reductions in fertility rates, as these may reflect more persistent and therefore more concerning patterns among adolescent girls. Provinces with non-significant trends were shown in grey. Sen's slope was calculated as a complementary estimate of the magnitude of annual change and is reported by province and age group in the Datasheet (supplementary material). The spatial analysis identified heterogeneous territorial patterns in the rate of decline of adolescent fertility among both early and late adolescents in Ecuador (Figure 6).

In early adolescents, territorial heterogeneity is particularly pronounced. Several Amazonian provinces, including Napo, Orellana, Pastaza, and Morona Santiago, appear in gray, indicating the absence of statistically significant monotonic trends. This suggests that in these provinces, fertility rates among girls showed insufficient evidence of a consistent decline during the study period. Among the provinces with significant trends, Esmeraldas (Coast), Sucumbíos (Amazonia), Manabí, Los Ríos (Coast), and Cañar (Sierra) exhibited more intense red tones, reflecting relatively slower declines. These provinces can be interpreted as areas where the reduction in early adolescent fertility has been insufficient or slower than in other regions of the country. In contrast, provinces such as Loja, El Oro, Guayas, and parts of the central Sierra showed lighter shades, corresponding to more negative tau values and therefore to stronger and more consistent reductions over time.

The pattern observed among adolescents aged 15–19 differed. In this group, almost all provinces showed statistically significant negative trends, indicating a more homogeneous nationwide decline in late adolescent fertility. However, important differences in the magnitude of these reductions persisted. Pastaza, in the Amazon region, stood out as the province with one of the slowest reductions in the country, shown by an intense red tone. Relatively weak declines were also observed in Esmeraldas, Manabí (Coast) and Napo (Amazonia), suggesting a persistent territorial presence of late adolescent fertility in these areas of the Amazon region and the Coast. In contrast, several provinces in the Sierra and the south of the country displayed lighter shades, indicating more pronounced and consistent declines.

From a territorial prioritization perspective, considering both groups, the findings identify the Amazon region and the north coast as notable because of the combination of historically high rates and slow or non-significant reductions in adolescent fertility rates.

### Social variables and adolescent fertility rates

Variables such as early union status, educational level and ethnicity were analyzed using data from the National Institute of Statistics and Census (INEC) and the Statistical Registry of Live Births. The information corresponds to 2022, the year of the most recent National Census.

The records identified 1,019 early adolescents (10–14 years) and 24,236 late adolescents (15–19 years) who gave birth while living in an early union. Adolescent fertility showed differences by union status in both age groups. Adolescents living in a union had higher fertility rates than those not cohabiting with a partner. Among early adolescents (10–14 years), the fertility rate for girls in a union was 283.06 births per 1,000 (95% CI: 265.94–300.98), compared with 2.07 per 1,000 (95% CI: 1.95–2.21) for those not in a union. A similar pattern was observed among late adolescents (15–19 years). This group in an early union had a fertility rate of 340.57 per 1,000 (95% CI: 336.30–344.89), compared with 31.61 per 1,000 (95% CI: 31.19–32.03) among those not in a union.

The following variables—education and ethnicity—are evaluated for the late adolescent group (15–19 years), which has more precise confidence intervals due to the larger population available for each social variable and its subcategories.

Fertility rates vary by educational level. Among late adolescents (15–19 years), rates were highest among those with basic education, at 201.82 births per 1,000 adolescents (IC95%: 198.70–204.95). Adolescents without education also showed elevated fertility, at 81.69 per 1,000 (IC95%: 70.79–92.59). Rates were lower among adolescents with secondary education (high school), at 49.46 per 1,000 (IC95%: 48.87–50.04), and among those attending university, at 15.30 births per 1,000 (IC95%: 14.59–16.01). Confidence intervals were narrow in the largest educational categories, reflecting precise estimates.

Adolescent fertility rates also vary by ethnicity. Among late adolescents (15–19 years), mestizo adolescents had the highest rate, at 69.46 births per 1,000 adolescents (95% CI: 68.78–70.14), followed by Indigenous adolescents at 50.72 per 1,000 (95% CI: 49.10–52.38). Afro-descendant adolescents had a rate of 31.35 per 1,000 (95% CI: 29.65–33.11). The lowest rates were recorded among White adolescents, at 16.62 per 1,000 (95% CI: 14.50–18.95).

## Discussion

As this is a descriptive and ecological study, the findings reveal temporal and territorial patterns. The differentiated patterns presented may guide targeted public health actions, support and reinforce the policy agenda for adolescent pregnancy prevention, even in light of a national reduction in fertility rates.

This study identified a decline in adolescent fertility rates in Ecuador between 2015 and 2024, across both early adolescents (10–14 years) and late adolescents (15–19 years). Declines were generally more consistent among late adolescents, while greater temporal variability was observed among the youngest.

Additionally, the results show that adolescent fertility is not spatially uniform. Higher rates were observed in Amazonia and the north Coast, whereas the lowest rates were observed in provinces of the Central Sierra region. Adolescents aged 15–19 years showed a more consistent and widespread decline across most provinces, whereas early adolescents exhibited greater territorial heterogeneity. In this group, high fertility rates were observed in the Amazon region and the province of Esmeraldas, while lower rates were observed in the Central Sierra. These findings suggest elevated fertility rates among early adolescents (10–14), despite the observed national decline.

From a territorial prioritization perspective, weaker or non-significant declines were observed in several provinces of Amazonia and the Coast, particularly Esmeraldas, Manabí, Los Ríos, Sucumbíos, Pastaza, and Napo, suggesting that these provinces may warrant continued monitoring and attention.

Differences were also observed by social variables. Adolescents in early unions have higher fertility rates than those not cohabiting with a partner in both age groups. Differences were likewise observed by educational level and ethnicity, with higher rates among late adolescents with lower educational attainment and among mestizo and indigenous populations.

The literature that may provide context for these findings shows that Ecuador is no exception to the concerning situation in Latin America and the Caribbean regarding adolescent pregnancy, a region where adolescent fertility remains high despite a decline, compared with other regions (14–16). Studies have identified several factors associated with adolescent pregnancy in Latin America, which stem mainly from exposure to violence, dysfunctional family problems, the age of the mother at first pregnancy, and the mother's level of education (17, 18). Furthermore, previous studies have discussed pregnancy among early adolescents in the context of sexual violence and child protection concerns (19, 20). In this regard, it is relevant to note that, under Ecuadorian law, sexual intercourse with a person under 14 years of age is classified as rape, since individuals below that age are not able to provide valid sexual consent (21). Although the present study did not measure violence, contextual data from the 2019 National Survey on Family Relationships and Gender-Based Violence against Women (ENVIGMU) illustrate a high prevalence of violence against women in Ecuador: 65 out of every 100 women aged 15 and older in Ecuador have experienced at least one act of violence in their lifetime, while 32 out of every 100 reported having suffered violence in the last 12 months (22). These figures, which are recommended for further analysis, may alert to broader situations of violence that could affect adolescent girls in Ecuador.

The higher fertility rates observed among adolescents in early unions are consistent with findings reported in previous studies. Plan International's 2025 report indicates that 22% of women aged 20–24 in Ecuador had entered into early unions before the age of 18. Furthermore, the report shows that 72% of girls (10–14 years) and adolescents (15–19 years) in early unions have at least one child (23). Additionally, 13% of adolescents reported experiencing violence and abuse from their partners, who are typically five or more years older (24, 25).

Identifying territories with persistently high rates or weaker temporal reductions poses specific challenges for public policy formulation. The territorial patterns identified in this study suggest that universal prevention strategies could be complemented by differentiated and territorially focused approaches. From a public health perspective, the findings highlight the need to strengthen territorial prioritization, ensure comprehensive protection for adolescents, facilitate access to comprehensive sexual and reproductive health services, and implement targeted monitoring.

For early adolescents (10–14 years), public policies may require special protective measures. This involves strengthening early detection systems for sexual abuse, improving safe reporting mechanisms, and ensuring a timely, coordinated response across health, education and social protection. For adolescents (15–19 years), policies should strengthen comprehensive sexuality education, ensure continuous and confidential access to modern contraceptive methods, and reduce administrative and cultural barriers within health services. This means ensuring the effective availability of adolescent-friendly services.

Considering adolescents who, due to various circumstances, have become mothers, education and health policies should include alternatives that allow them to continue their studies; this means school retention and psychosocial support so that cycles of social vulnerability do not persist due to a lack of professional skills and the abrupt stagnation of life projects.

One of the main contributions of this study is the use of temporal and spatial analyses and the consideration of two age groups, using national population data over a ten-year period. In this way, it is possible to identify different epidemiological patterns that might remain hidden when adolescent fertility is analyzed as a single age category. This approach is particularly relevant in Latin America, a region with economic and social disparities. The use of territorial and temporal analyses can contribute to the strengthening and continuous monitoring of adolescent fertility prevention policies, facilitating the implementation of strategies better suited to the local and age-specific realities of female adolescents.

Some limitations should be considered. First, the analysis was based on aggregated provincial-level data and followed a descriptive ecological design; therefore, it is not possible to draw individual-level inferences or establish causal relationships between adolescent fertility rates and the contextual factors discussed in the literature. As a result, the associations described by social variables (union status, education, ethnicity) reflect group-level patterns and should not be interpreted as individual predictors of adolescent fertility. Second, the quality and completeness of administrative records may vary across provinces and over time, particularly in rural, dispersed, or hard-to-reach areas. Third, because the analysis relied on live birth records, it did not capture pregnancies that ended in miscarriage, stillbirth, or induced abortion. These results should therefore be interpreted as indicative of temporal and territorial patterns that may support public health surveillance and prioritization.

Future research integrating multi-source data would be needed to establish social determinants and associations with poverty, gender-based violence, and lack of education, as well as to conduct a more in-depth analysis of ethnic minorities. Research could focus on the Amazon Region and the North Coast of Ecuador, identified in this study as areas of attention. This approach may require both quantitative and qualitative studies, which would provide greater clarity on the social processes shaping pregnancy among girls and adolescents in Ecuador, as well as on the particular strategies needed.

## Conclusions

Following a descriptive ecological design, this work integrates temporal and spatial analyses, disaggregated by age group, using national data over a decade. The differentiation between early and late adolescents allowed the identification of distinct temporal and territorial patterns that might not be visible in conventional analyses of adolescent fertility.

Although adolescent fertility in Ecuador declined nationally between 2015 and 2024, subnational analyses revealed heterogeneous temporal and territorial patterns, including differences in the magnitude and consistency of fertility declines across provinces and age groups. Late adolescents experienced more consistent and homogeneous reductions across provinces, whereas greater spatial heterogeneity persisted among early adolescents, including provinces with non-significant trends or slower declines. The highest rates and weakest reductions were mainly concentrated in Amazonian and the North Coast through the study period.

In addition, the exploration of social variables such as union status, educational level, and ethnicity might suggest that higher rates are observed among adolescents living in a union, with lower educational attainment, and those from mestizo or indigenous origins.

The findings underscore the importance of subnational and age-differentiated monitoring of adolescent fertility and may contribute to territorial surveillance and evidence-informed public health planning in Ecuador and other Latin American settings.

## Data Availability

The original contributions presented in the study are included in the article/Supplementary Material, further inquiries can be directed to the corresponding author.
