# Supplementary material for: Beyond national fertility rates: unmasking territorial differences in adolescent pregnancy in Ecuador
Source: Front Reprod Health. 2026 Jul 22;8:1863795. doi: 10.3389/frph.2026.1863795 (PMC13437521; doi:10.3389/frph.2026.1863795)
Supplement: Supplementary file 1 [file Table1.docx]

**Table S.1** Temporal trends in adolescent fertility rates among girls aged 10–14 years, by province. Ecuador, 2015–2024

| **Province** | **Mean annual percent change (%)** | **Mann–Kendall tau** | **Mann–Kendall p-value** | **Sen's slope (births/1,000/year)** | **Sen's slope p-value** | **Significance** |
| --- | --- | --- | --- | --- | --- | --- |
| Azuay | -4.81 | -0.60 | 0.0200 | -0.09 | 0.0200 | * |
| Bolívar | 7.61 | 0.11 | 0.7205 | 0.02 | 0.7205 |  |
| Cañar | -1.59 | -0.56 | 0.0318 | -0.21 | 0.0318 | * |
| Carchi | -2.79 | -0.24 | 0.3711 | -0.05 | 0.3711 |  |
| Cotopaxi | -0.40 | -0.42 | 0.1074 | -0.05 | 0.1074 |  |
| Chimborazo | 18.21 | 0.11 | 0.7205 | 0.01 | 0.7205 |  |
| El Oro | -1.12 | -0.69 | 0.0073 | -0.12 | 0.0073 | ** |
| Esmeraldas | -4.48 | -0.51 | 0.0491 | -0.24 | 0.0491 | * |
| Guayas | -6.97 | -0.91 | 0.0003 | -0.15 | 0.0003 | *** |
| Imbabura | -2.51 | -0.64 | 0.0123 | -0.11 | 0.0123 | * |
| Loja | 3.49 | -0.16 | 0.5915 | -0.03 | 0.5915 |  |
| Los Ríos | -5.57 | -0.60 | 0.0200 | -0.23 | 0.0200 | * |
| Manabí | -5.04 | -0.60 | 0.0200 | -0.16 | 0.0200 | * |
| Morona Santiago | 0.65 | -0.42 | 0.1074 | -0.20 | 0.1074 |  |
| Napo | 16.58 | 0.24 | 0.3711 | 0.21 | 0.3711 |  |
| Pastaza | 3.15 | -0.33 | 0.2105 | -0.18 | 0.2105 |  |
| Pichincha | -5.16 | -0.64 | 0.0123 | -0.10 | 0.0123 | * |
| Tungurahua | 0.33 | -0.42 | 0.1074 | -0.04 | 0.1074 |  |
| Zamora Chinchipe | 4.77 | -0.16 | 0.5915 | -0.04 | 0.5915 |  |
| Galápagos | 24.87 | 0.25 | 0.4544 | 0.01 | 0.4544 |  |
| Sucumbíos | -2.87 | -0.60 | 0.0200 | -0.30 | 0.0200 | * |
| Orellana | 1.60 | -0.33 | 0.2105 | -0.33 | 0.2105 |  |
| Santo Domingo | -6.59 | -0.82 | 0.0013 | -0.27 | 0.0013 | ** |
| Santa Elena | -4.63 | -0.42 | 0.1074 | -0.07 | 0.1074 |  |
|  |  |  |  |  |  |  |
| ** p < 0.05; ** p < 0.01; *** p < 0.001. Provinces with non-significant Mann–Kendall trends (p ≥ 0.05) have no significance symbol. Sen's slope represents the median annual rate of change in the adolescent fertility rate (births per 1,000 adolescents) over 2015–2024.* | | | | | | |

**TABLE S2.** Temporal trends in adolescent fertility rates among adolescents aged 15–19 years, by province. Ecuador, 2015–2024

| **Province** | **Mean annual percent change (%)** | **Mann–Kendall tau** | **Mann–Kendall p-value** | **Sen's slope (births/1,000/year)** | **Sen's slope p-value** | **Significance** |
| --- | --- | --- | --- | --- | --- | --- |
| Azuay | -8.49 | -1.00 | 0.0001 | -4.85 | 0.0001 | *** |
| Bolívar | -5.84 | -0.82 | 0.0013 | -3.24 | 0.0013 | ** |
| Cañar | -7.52 | -0.73 | 0.0042 | -7.62 | 0.0042 | ** |
| Carchi | -7.38 | -0.96 | 0.0002 | -4.42 | 0.0002 | *** |
| Cotopaxi | -6.91 | -0.96 | 0.0002 | -3.55 | 0.0002 | *** |
| Chimborazo | -4.92 | -0.87 | 0.0007 | -2.66 | 0.0007 | *** |
| El Oro | -7.37 | -0.82 | 0.0013 | -4.36 | 0.0013 | ** |
| Esmeraldas | -4.15 | -0.64 | 0.0123 | -3.75 | 0.0123 | * |
| Guayas | -8.34 | -0.96 | 0.0002 | -4.84 | 0.0002 | *** |
| Imbabura | -8.07 | -0.96 | 0.0002 | -4.02 | 0.0002 | *** |
| Loja | -6.03 | -0.96 | 0.0002 | -3.24 | 0.0002 | *** |
| Los Ríos | -6.49 | -0.82 | 0.0013 | -5.05 | 0.0013 | ** |
| Manabí | -4.94 | -0.64 | 0.0123 | -3.90 | 0.0123 | * |
| Morona Santiago | -4.87 | -0.82 | 0.0013 | -3.53 | 0.0013 | ** |
| Napo | -1.92 | -0.69 | 0.0073 | -2.27 | 0.0073 | ** |
| Pastaza | -5.05 | -0.60 | 0.0200 | -4.23 | 0.0200 | * |
| Pichincha | -9.36 | -0.96 | 0.0002 | -4.44 | 0.0002 | *** |
| Tungurahua | -7.67 | -1.00 | 0.0001 | -4.24 | 0.0001 | *** |
| Zamora Chinchipe | -5.58 | -0.73 | 0.0042 | -4.30 | 0.0042 | ** |
| Galápagos | -5.25 | -0.47 | 0.0736 | -2.79 | 0.0736 |  |
| Sucumbíos | -6.43 | -0.82 | 0.0013 | -5.78 | 0.0013 | ** |
| Orellana | -7.50 | -0.82 | 0.0013 | -7.74 | 0.0013 | ** |
| Santo Domingo | -7.31 | -0.82 | 0.0013 | -5.24 | 0.0013 | ** |
| Santa Elena | -6.22 | -0.87 | 0.0007 | -3.78 | 0.0007 | *** |
|  |  |  |  |  |  |  |
| ** p < 0.05; ** p < 0.01; *** p < 0.001. Provinces with non-significant Mann–Kendall trends (p ≥ 0.05) have no significance symbol. Sen's slope represents the median annual rate of change in the adolescent fertility rate (births per 1,000 adolescents) over 2015–2024.* | | | | | | |
