# Supplementary material for: Beyond national fertility rates: unmasking territorial differences in adolescent pregnancy in Ecuador
Source: Front Reprod Health. 2026 Jul 22;8:1863795. doi: 10.3389/frph.2026.1863795 (PMC13437521; doi:10.3389/frph.2026.1863795)
Supplement: Supplementary file 2 [file Table2.docx]

**TABLE S2.** Temporal trends in adolescent fertility rates among adolescents aged 15–19 years, by province. Ecuador, 2015–2024

| **Province** | **Mean annual percent change (%)** | **Mann–Kendall tau** | **Mann–Kendall p-value** | **Sen's slope (births/1,000/year)** | **Sen's slope p-value** | **Significance** |
| --- | --- | --- | --- | --- | --- | --- |
| Azuay | -8.49 | -1.00 | 0.0001 | -4.85 | 0.0001 | *** |
| Bolívar | -5.84 | -0.82 | 0.0013 | -3.24 | 0.0013 | ** |
| Cañar | -7.52 | -0.73 | 0.0042 | -7.62 | 0.0042 | ** |
| Carchi | -7.38 | -0.96 | 0.0002 | -4.42 | 0.0002 | *** |
| Cotopaxi | -6.91 | -0.96 | 0.0002 | -3.55 | 0.0002 | *** |
| Chimborazo | -4.92 | -0.87 | 0.0007 | -2.66 | 0.0007 | *** |
| El Oro | -7.37 | -0.82 | 0.0013 | -4.36 | 0.0013 | ** |
| Esmeraldas | -4.15 | -0.64 | 0.0123 | -3.75 | 0.0123 | * |
| Guayas | -8.34 | -0.96 | 0.0002 | -4.84 | 0.0002 | *** |
| Imbabura | -8.07 | -0.96 | 0.0002 | -4.02 | 0.0002 | *** |
| Loja | -6.03 | -0.96 | 0.0002 | -3.24 | 0.0002 | *** |
| Los Ríos | -6.49 | -0.82 | 0.0013 | -5.05 | 0.0013 | ** |
| Manabí | -4.94 | -0.64 | 0.0123 | -3.90 | 0.0123 | * |
| Morona Santiago | -4.87 | -0.82 | 0.0013 | -3.53 | 0.0013 | ** |
| Napo | -1.92 | -0.69 | 0.0073 | -2.27 | 0.0073 | ** |
| Pastaza | -5.05 | -0.60 | 0.0200 | -4.23 | 0.0200 | * |
| Pichincha | -9.36 | -0.96 | 0.0002 | -4.44 | 0.0002 | *** |
| Tungurahua | -7.67 | -1.00 | 0.0001 | -4.24 | 0.0001 | *** |
| Zamora Chinchipe | -5.58 | -0.73 | 0.0042 | -4.30 | 0.0042 | ** |
| Galápagos | -5.25 | -0.47 | 0.0736 | -2.79 | 0.0736 |  |
| Sucumbíos | -6.43 | -0.82 | 0.0013 | -5.78 | 0.0013 | ** |
| Orellana | -7.50 | -0.82 | 0.0013 | -7.74 | 0.0013 | ** |
| Santo Domingo | -7.31 | -0.82 | 0.0013 | -5.24 | 0.0013 | ** |
| Santa Elena | -6.22 | -0.87 | 0.0007 | -3.78 | 0.0007 | *** |
|  |  |  |  |  |  |  |
| ** p < 0.05; ** p < 0.01; *** p < 0.001. Provinces with non-significant Mann–Kendall trends (p ≥ 0.05) have no significance symbol. Sen's slope represents the median annual rate of change in the adolescent fertility rate (births per 1,000 adolescents) over 2015–2024.* | | | | | | |
